# New biosourced AA and AB monomers from 1,4:3,6-dianhydrohexitols, Isosorbide, Isomannide, and Isoidide

**DOI:** 10.1080/15685551.2016.1239175

**Published:** 2016-10-23

**Authors:** Asma Saadaoui, Raouf Medimagh, Sylvain Marque, Damien Prim, Saber Chatti, Herve Casabianca, Mongia Said Zina

**Affiliations:** ^a^ Laboratoire des Substances Naturelles (LR10INRAP02), Institut National de Recherche et d’Analyse Physico-chimique (INRAP), Ariana, Tunisia; ^b^ Faculté des Sciences de, Université de Tunis El Manar, Tunis, Tunisia; ^c^ Institut des Sciences Analytiques, UMR5280, CNRS, Université de Lyon 1, ENS-Lyon, Villeurbanne, France; ^d^ Institut Lavoisier de Versailles (ILV) UMR CNRS 8180, Université de Versailles-St-Quentin (UVSQ), Versailles Cedex, France

**Keywords:** 1,4:3,6-Dianhydrohexitols, Isosorbide, biosourced, AB monomers, semicrystalline, poly(ether)esters

## Abstract

In the present work, we propose the synthesis of a new family of sugar derived 1,4:3,6-dianhydrohexitol based AA/AB-type monomers. Unprecedented diacids based on Isomannide and Isoidide were elaborated with high yields and showed interestingly high melting point ranges (240–375 °C). Optimization of reaction conditions (temperature, time of reaction, and reactant ratios) has been investigated to synthesize the key intermediate of a set of AB monomers with acid, ester, and acid chloride functionalities. Isosorbide based ether benzoic acid AB monomer was polymerized and characterized by NMR and DSC techniques. The results show a semicrystalline behavior of the obtained polymer thanks to the controlled stereoregular arrangement of the AB starting monomer.

## 1. Introduction

Pushed by concerns about the availability of petrochemical raw materials and the necessity for a sustainable society, bio-based materials became viable alternatives to existing petrochemicals. The most abundant carbohydrates attract widespread attention in both academic and industrial communities for the production of chemicals, fuels, and energy. However, a major disadvantage of using carbohydrates directly in industrial processes is their limited thermal stability due to the presence of different reactive functional groups.

The development of new monomers for the synthesis of original materials is predominantly motivated by the need to overcome the main shortcomings of composites. Therefore, to obtain mono and bi-functional monomers the depolymerization of carbohydrates is a practical strategy,[1,2] providing building blocks such as furan-25-dicarboxylic acid,[3–5] succinic acid, [6] and 1,4:3,6-dianhydrohexitols (DAH).[7,8] One of the most explored fields concerning the application of DAH is polymers, mostly referring to step‐growth polymers. A first review was published by Braun and Bergman in 1992, in which they discussed the early developments of DAH‐based polyesters, polycarbonates and polyurethanes. Later on, Kricheldorf presented another progress report [9] mainly focusing on DAH‐derived polyesters with special optical and liquid crystalline properties.[10,11] Recently, an excellent review was published by Fenouillot [7] which covers not only the most important and latest results on this subject but also the commercial applicability as well as the industrial potential of DAH‐derived monomers. However, some limitations due to a lack of stability and/or reactivity gave rise to several attempts in order to significantly broaden their application fields.[12] Modified DAH, displaying diamine,[13,14] amines,[15] acids,[14] or extended primary alcohols [16] found application in polyamides,[13] polyesters,[8,12,17] poly(ether)ketones [18] and poly(ether)sulfones.[19]

Among our recent efforts in this field we reported the synthesis of three diamines based on Isosorbide (**1),** Isomannide (**2),** and Isoidide (**3)** which were suitably used for the synthesis of polyamides,[20] and poly(ether)ureas.[21] Poly(ether)esteramides, as for them, were obtained directly from aminoalcohols [22] based on Isosorbide and Isomannide or from novel bis amide-diols building blocks.[23]

**Scheme 1.**
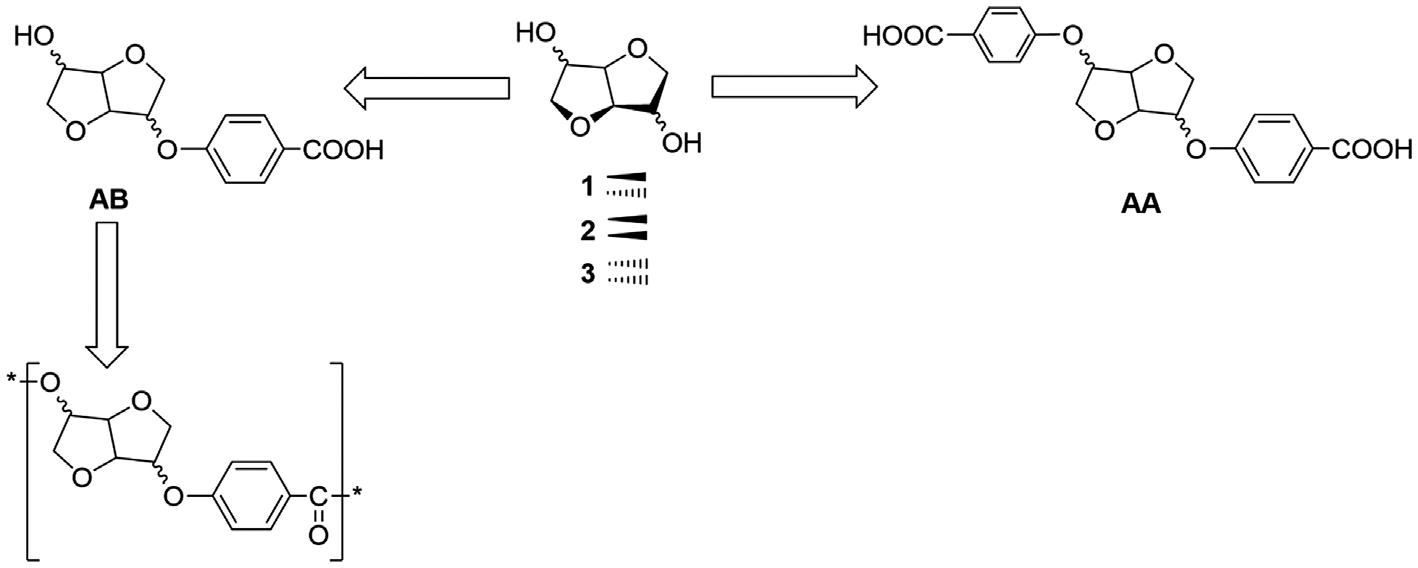
Synthetic route of novel monomers issued from dianhydrohexitols.

In another context, Jaffe published appealing AB type monomers containing phenyl moieties with both ester and ether linkers.[24] The DAH backbone was Isosorbide type for the former and Isoidide type for the latter. This strategy allowed them to obtain semicrystalline thermoplastics due to the controlled orientation of the hydroxyl functions for the former monomer. In this context, we propose herein a strategy to develop a new platform of DAH-based versatile monomers with a variety of functional groups. These unprecedented bio-sourced monomers exhibit AA and AB unsymmetrical functions. They could serve as monomers or precursors suitable for step‐growth (Scheme 1).

We present herein new monomers from the three DAH isomers starting from versatile benzonitrile compounds. Their hydrolysis led quantitatively to the corresponding diacids. In another side, dissymmetrical AB type monomers starting from protected Isosorbide or Isomannide reported for the first time are described. Their characterization and thermal properties as well as polymerization example are discussed in the present study.

## 2. Experimental

### 2.1. Materials and methods

Isosorbide was purchased from Alfa Aesar (Karleshrue, Germany), Isomannide and Isoidide were purchased from Sigma Aldrich (Milwaukee, WI, USA), previously crystallized from acetone and dried under high vacuum. 1-fluoro-4-benzonitrile (99%), 1,4,7,10,13,16-hexaoxacyclooctadecane (18-Crown-6), activated palladium supported on charcoal (10%), and thionyl chloride were purchased from Sigma-Aldrich (St Louis, MO, USA). KOH (Normapur) was purchased from Prolabo (Paris, France). Unless otherwise mentioned, all the reactants were used as received. The solvents were distilled over CaH_2_ and placed on molecular sieves.

1D and 2D NMR techniques were recorded at 300 and 500 MHz (Bruker WP 250). The chemical shifts are given in ppm. Differential Scanning Calorimetric data were obtained using DSC131 (SETARAM). DSC measurements were conducted with a heating and cooling rate at 10 °C/min. The first heating cycle was conducted from room temperature to 150 °C. Then samples were cooled down to room temperature. Then, a second heating scan was conducted (RT to 500 °C for the diacid) (RT to 300 °C for the AB monomers) (RT to 250 °C for the polymer). The *T*_m_ and *T*_g_ values were determined from the second DSC heating scan. Sample weights of about 10−15 mg were used in these experiments.

### 2.2. Monomers synthesis

#### 2.2.1. Synthesis of dinitrile compounds

Dianhydrohexitol (10 mmol, 1.46 g), finely ground potassium hydroxide KOH (2.6 eq, 26 mmol, 1.47 g), 18-crown-6 (10%, 1 mmol, 0.265 g), and *p*-fluorobenzonitrile (2.5 eq, 25 mmol, 3.03 g) were placed in a cylindrical reactor equipped with a mechanical stirring. The reaction media was warmed gradually to 170 °C and stirring was maintained for4 h. The mixture was then cooled to room temperature and the crude was diluted with dichloromethane and filtered through a glass filter with celite and the residue was rinsed with dichloromethane. The solvent was then removed by rotary evaporator. The crude product was purified by flash chromatography on silica gel using a gradient eluent pentane / ethyl acetate: 70/30 (or by precipitation in cold MeOH).

##### 2.2.1.1. 1,4:3,6-dianhydro-25-di-O-(4-cyanophenyl)-D-sorbitol (**11**)

Yellowish powder, 90% yield; ^1^H NMR (300 MHz, CDCl_3_): *δ* (ppm) 7.63–7.59 (dd, 4H, H9, *J* = 8.7 Hz, *J*’ = 2.2 Hz); 7.05–6.99 (m, 4H, H8); 5.05 (t, 1H, H4, *J* = 10 Hz, *J*’ = 5.2 Hz); 4.89–4.84 (m, 2H, H5 + H2); 4.64 (d, 1H, H3, *J* = 5.2 Hz); 4.19–4.02 (m, 4H, H1 + H6); ^13^C NMR (75 MHz, CDCl_3_): *δ* (ppm) 161.06 (C7); 160.06 (C7); 134.16 (C9); 133.98 (C9); 118.93 (CN); 118.84 (CN); 115.82 (C8); 115.74 (C8); 104.91 (C10); 104.79 (C10); 8611 (C3); 8155(C2); 8150 (C5); 7751 (C4); 73.23 (C1); 71.63 (C6); IR (KBr): cm^−1^ 2964, 2890, 2226, 1602, 1502, 1382, 1173, 1097, 817; HR-MS m/z for C_20_H_17_N_2_O_4_ [M + H]^+^: calcd: 349.1188, found 349.1415; *T*_m_ = 130.6–137.9 °C.

##### 2.2.1.2. 1,4:3,6-dianhydro-25-di-O-(4-cyanophenyl)-D-mannitol (**21**)

Brown powder, 84% yield; ^1^H NMR (300 MHz, CDCl_3_): *δ* (ppm) 7.60–7.57 (d, 4H, H9, *J* = 9 Hz); 7.05–7.02 (d, 4H, H8, *J* = 9 Hz); 4.91–4.82 (m, 2H, H5); 4.85–4.82 (m, 2H, H4); 4.13–4.01 (m, 4H, H1). ^13^C NMR (75 MHz, CDCl_3_): *δ* (ppm) 161.00 (C7); 133.95 (C9); 118.90 (CN); 115.69 (C8); 104.65 (C10); 80.77 (C4); 77.05 (C5); 71.45 (C6); IR (KBr): cm^−1^ 2931, 2865, 2223, 1606, 1507, 1302, 1173, 1063, 840; HR-MS m/z for C_20_H_17_N_2_O_4_ [M + H]+: calcd 3,491,188, found 349.1186; *T*_m_ = 178.9–184.4 °C.

##### 2.2.1.3. 1,4:3,6-dianhydro-25-di-O-(4-cyanophenyl)-D-iditol (**31**)

Yellowish powder, 92% yield; ^1^H NMR (300 MHz, CDCl_3_): *δ* (ppm) 7.62–7.60 (d, 4H, H9, *J* = 8.9 Hz); 7.01–6.99 (d, 4H, H8, *J* = 8.8 Hz); 489 (dd, 2H, H2,*J* = 3.8 Hz, *J* = 2.1 Hz); 4.77–4.79 (m, 2H, H3); 4.25–4.07 (m, 4H, H1); ^13^C NMR (75 MHz, CDCl_3_): *δ* (ppm) 160.03 (C7); 134.14 (C9); 118.77 (CN); 115.79 (C8); 104.96 (C10); 85.56 (C3); 81.13 (C2); 72.16 (C1); IR (KBr): cm^−1^ 2922, 2869, 2222, 1603, 1499, 1296, 1092, 841; HR-MS m/z for C_20_H_17_N_2_O_4_ [M + H]^+^: calcd 349.1188, found 349.1190; *T*_m_ = 160.1–165.8 °C.

#### 2.2.2. Synthesis of diacid compounds

The dinitrilphenyl compound (1.44 mmol, 0.5 g) was suspended in a mixture of EtOH/H_2_O 50/50 (20 mL) with KOH (11 eq, 15.84 mmol, 0.89 g) in a two-necked round bottom flask and stirred magnetically. The medium was warmed to 100 °C and the reaction was refluxed for 48 h. After completion (TLC control), the mixture was cooled to room temperature and a few drops of HCl (cc) were added and a precipitate began to be formed. The addition was continued to reach acidic pH (pH = 2–3). The precipitate is then filtered, washed with water and vacuum dried at 50 °C, until constant weight. The diacid was recovered as a pure white crystalline solid.

##### 2.2.2.1. 4,4’-(((3R,3aR,6S,6aR)-hexahydrofuro[3,2-b]furan-3,6-diyl)bis(oxy))dibenzoic acid (**12**)

White powder, 98% yield; ^1^H NMR (500 MHz, DMSO-*d6*): *δ* (ppm) 7.91–7.86 (m, 4H, H9); 7.10–7.03 (m, 4H, H8); 5.05–5.01 (m, 2H, H2 + H5); 5.00 (d, 1H, H4, *J* = 3.5 Hz); 458 (d, 1H, H3, *J* = 4.4 Hz); 404–400 (m, 2H, H1 + H6); 3.92–3.89 (m, 2H, H1 + H6); ^13^C NMR (75 MHz, CDCl_3_): *δ* (ppm 167.19 (COOH); 167.11 (COOH); 161.67 (C7); 160.42 (C7); 131.68 (C9); 131.43 (C9); 123.80 (C10); 123.39 (C10); 115.18 (C8); 114.96 (C8); 85.94 (C3); 8143 (C2); 80.92 (C4); 77.16 (C5); 72.78 (C1); 70.98 (C6); IR: cm^−1^ 2941, 2665, 1676, 1596, 1240, 1170, 1083, 841; *T*_m_ = 240–300 °C*.*

##### 2.2.2.2. 4,4’-(((3R,3aR,6R,6aR)-hexahydrofuro[3,2-b]furan-3,6-diyl)bis(oxy))dibenzoic acid (**22**)

White powder, 95% yield; ^1^H NMR (500 MHz, DMSO-*d6*): δ (ppm) 7.88–7.87 (d, 4H, H9, *J* = 8.8 Hz); 7.13–7.11 (d, 4H, H8, *J* = 8.8 Hz); 5.01–4.94 (m, 2H, H5); 4.91–4.90 (m, 2H, H4); 4.03–4.00 (m, 2H, H1); 3.81–3.78 (m, 2H, H1). ^13^C NMR (125 MHz, CDCl_3_): *δ* (ppm) 167.00 (COOH); 161.47 (C7); 131.29 (C9); 123.25 (C10); 114.83 (C8); 80.22 (C4); 76.73 (C5); 70.65 (C6); IR: cm^−1^ 2941, 2665, 1676, 1596, 1240, 1170, 1083, 841; *T*_m_ = 300–375 °C.

##### 2.2.2.3. 4,4’-(((3S,3aR,6S,6aR)-hexahydrofuro[3,2-b]furan-3,6-diyl)bis(oxy))dibenzoic acid (32)

White powder, 96% yield; ^1^H NMR (500 MHz, DMSO-*d6*): *δ* (ppm) 7.91–7.89 (m, 4H, H9); 7.13–7.11 (m, 4H, H8); 5.04 (dd, 2H, H2, *J* = 3.8 Hz, *J* = 1.6 Hz); 4.72 (s, 2H, H3); 4.13–4.10 (dd, 2H, H1, *J* = 10.4 Hz, *J* = 4.1 Hz); 3.98–3.96 (dd, 2H, H1, *J* = 10.4 Hz, *J* = 1.6 Hz); ^13^C NMR (125 MHz, CDCl_3_): *δ* (ppm) 166.85 (COOH); 160.21 (C7); 131.46 (C9); 123.67 (C10); 115.06 (C8); 85.24 (C3); 80.84 (C2); 71.56 (C1); IR: cm^−1^ 2941, 2665, 1676, 1596, 1240, 1170, 1083, 841; *T*_m_ = 280–315 °C.

#### 2.2.3. Synthesis of mono-nitrile compounds

**1a** or **2a** [22] (4.23 mmol, 1 g), finely ground potassium hydroxide KOH (1.2 eq, 5 mmol, 0.28 mg), 18-crown-6 (10%, 0.112 g),and *p*-fluorobenzonitrile (1.1 eq, 4.65 mmol, 0.564 g) were placed in a cylindrical reactor equipped with a mechanical stirring. The reaction media was warmed gradually to 170 °C and stirring was maintained during 4 h. The mixture was then cooled to room temperature and the crude was diluted with dichloromethane and filtered through a glass filter with celite and the residue was rinsed with dichloromethane. The solvent was then removed by rotary evaporator. The crude product was purified by flash chromatography on silica gel using a gradient eluent pentane / ethyl acetate: 60/40 (or by precipitation in cold MeOH).

##### 2.2.3.1. 4-(((3R,3aR,6S,6aR)-6-(benzyloxy)hexahydrofuro[3,2-b]furan-3-yl)oxy)benzonitrile (**1b**)

Yellowish powder, 86% yield; ^1^H NMR (300 MHz, CDCl_3_): *δ* (ppm) 7.61–7.58 (m, 2H, H9); 7.37–7.30 (m, 5H, Hph); 7.03–7.00 (m, 2H, H8); 4.97 (t, 1H, H4, *J* = 5 H); 4.84–4.79 (q, 1H, H5, *J* = 15.6 Hz, *J*’ = 5.2 Hz); 4.62 (d, 1H, H3, *J* = 4.9 Hz); 4.58 (s, 2H, CH_2_); 4.14 (d, 1H, H2, *J* = 3.3 Hz); 4.05–3.89 (m, 4H, H1 + H6); ^13^C NMR (75 MHz, CDCl_3_): *δ* (ppm) 161.25 (C7); 137.38 (Ca); 133.96 (C9); 128.49 (Cb); 127.91 (Cd); 127.69 (Cc); 119.03 (CN); 115.78 (C8); 104.60 (C10); 86.78 (C3); 82.97 (C2); 80.88 (C4); 77.86 (C5); 73.26 (C1); 71.43 (C6); 70.99 (CH_2_); IR (KBr): cm^−1^ 3027, 2996, 2913, 2220, 1601, 1507, 1219, 1173, 966; HR-MS m/z for C_20_H_20_NO_4_ [M + H]^+^: calcd 338.1392, found 338.1396; *T*_m_ = 84.9–86.1 °C.

##### 2.2.3.2. 4-(((3R,3aR,6R,6aR)-6-(benzyloxy)hexahydrofuro[3,2-b]furan-3-yl)oxy)benzonitrile (**2b**)

Yellowish powder, 55% yield; ^1^H NMR (300 MHz, CDCl_3_): *δ* (ppm) 7.62–7.57 (d, 2H, *J* = 8.8 Hz, H9); 7.34 (s, 5H, Hph); 7.04–6.99 (d, 2H, H8, *J* = 8.8 Hz); 4.99–4.94 (m,1H, H4); 4.85–4.77 (q, 1H, H5, *J* = 10.4 Hz, *J* = 5.2 Hz); 4.62–4.61 (d, 1H, H3, *J* = 4.9 Hz); 4.58 (s, 2H, CH_2_); 4.13 (s, 1H, H2); 4.03–3.87 (m, 4H, H1 + H6); ^13^C NMR (75 MHz, CDCl_3_): *δ* (ppm) 161.15 (C7); 137.31 (Ca); 133.86 (C9); 128.33 (Cb); 127.81 (Cd); 127.59 (Cc); 118.93 (CN); 115.70 (C8); 104.58 (C10); 86.71 (C3); 82.95 (C2); 80.78 (C4); 77.80 (C5); 73.20 (C1); 71.32 (C6); 70.94 (CH_2_); IR (KBr): cm^−1^ 3100, 2986, 2226, 1615, 1572, 1382, 1173, 1020, 816; HR-MS m/z for C_20_H_20_NO_4_ [M + H]^+^: calcd 338.1392, found 338.1391; *T*_m_ = 84.4–85.9 °C.

#### 2.2.4. General debenzylation method

**1b** or **2b** (1.81 mmol, 650 mg) was dissolved in AcOEt (25 mL) and 20% of Pd/C (0.130 g) was added. The reaction mixture was reduced under hydrogen pressure (4 bars) at 40 °C. After 36 h, the crude was filtered over thick filter paper and rinsed with acetonitrile (25 mL). After evaporation under vacuum, the product was purified by flash chromatography on silica gel using a gradient eluent pentane / ethyl acetate: 50/50.

### 2.2.4.1. 4-(((3R,3aR,6S,6aR)-6-hydroxyhexahydrofuro[3,2-b]furan-3-yl)oxy)benzonitrile (**1c**)

yellow powder, 85% yield; ^1^H NMR (300 MHz, CDCl_3_): *δ* (ppm) 7.61–7.57 (d, 2H, H9, *J* = 9 Hz); 7.02–6.98 (d, 2H, H8, *J* = 8 Hz); 4.98–4.90 (q, 1H, H4, *J* = 10 Hz, *J* = 5 Hz); 4.84–4.77 (m, 2H, H5); 4.57 (t, 1H, H3, *J* = 5.9 Hz); 4.35 (s, 1H, H2); 3.99–3.83 (m, 4H, H1 + H6); ^13^C NMR (75 MHz, CDCl_3_): *δ* (ppm) 161.19 (C7); 134.08 (C9); 118.96 (CN); 115.82 (C8); 104.82 (C10); 81.82 (C3); 80.88 (C4); 80.79 (C5); 75.30 (C2); 71.76 (C1); 70.15 (C6); IR (KBr): cm^−1^ 3400, 2881, 2222, 1605, 1507, 1255, 1108, 833; HR-MS m/z for C_13_H_13_NO_4_ [M + H]^+^: calcd 247.0845, found 247.0844; *T*_m_ = 79.4–81.9 °C.

#### 2.2.5. Fractionwise addition method with Isommanide

Isomannide (0.69 mmol, 0.1 g), finely ground potassium hydroxide KOH (0.6 eq, 0.414 mmol, 0.23 g), 18-crown-6 (10%, 0.018 g), *p*-fluorobenzonitrile (0.6 eq, 0.414 mmol, 0.05 g) were placed in a cylindrical reactor equipped with a mechanical stirring. The reaction media was warmed gradually to 150 °C and stirring was maintained during 5 h. The mixture was then cooled to room temperature. The crude was diluted with dichloromethane and filtered through a glass filter with celite and the residue was rinsed with dichloromethane. The solvent was then removed by rotary evaporator. The crude product was purified by flash chromatography on silica gel using a gradient eluent pentane / ethyl acetate: 50/50.

##### 2.2.5.1. 4-(((3R,3aR,6R,6aR)-6-hydroxyhexahydrofuro[3,2-b]furan-3-yl)oxy)benzonitrile (**2c**)

White powder, 84% yield; ^1^H NMR (300 MHz, CDCl_3_): *δ* (ppm) 7.63–7.59 (m, 4H, H9); 7.06–7.01 (m, 4H, H8); 4.90–4.83 (m, 2H, H5 + H4); 4.55–4.53 (m, 1H, H3); 4.31–4.26 (m, 1H, H2); 4.18–3.74 (m, 4H, H1 + H6); ^13^C NMR (75 MHz, CDCl_3_): *δ* (ppm) 161.09 (C7); 134.02 (C9); 118.94 (CN); 115.76 (C8); 104.80 (C10); 81.78 (C3); 80.85 (C4); 80.77 (C5); 75.23 (C2); 71.74 (C1); 70.12 (C6); IR (KBr): cm^−1^ 3500, 2913, 2220, 1601, 1507, 1456, 1220, 1044, 968; HR-MS m/z for C13H13NO4 [M + H]^+^: calcd 247.0845, found 247.0842; *T*_m_ = 119.6–125.1 °C.

#### 2.2.6. Synthesis of the monoacids

**1b** or **2b** (2.96 mmol, 1 g) was suspended in a mixture of EtOH/ H_2_O 50/50 (20 mL) with KOH (11 eq, 32.6 mmol, 1.83 g) in a two-necked round bottom flask and stirred magnetically. The medium was warmed to 100 ^°^C and the reaction was refluxed for 48 h. After completion (TLC control), the mixture was cooled to room temperature and a few drops of HCl (cc) were added up to precipitate formation. The addition was continued until acidic pH was reached (pH = 2–3). The precipitate was then filtered, washed thoroughly with water and vacuum dried at 50 ^°^C until constant weight. The acid was recovered as a pure white crystalline solid.

##### 2.2.6.1. 4-(((3R,3aR,6S,6aR)-6-(benzyloxy)hexahydrofuro[3,2-b]furan-3-yl)oxy)benzoic acid (**1d**)

White powder, 95% yield; ^1^H NMR (500 MHz, DMSO-*d*6): *δ* (ppm) 7.87–7.71 (m, 2H, H9); 7.34–7.28 (m, 5H, Hph); 7.15–7.05 (m, 2H, H8); 5.00–4.91 (m, 1H, H4 + H5); 4.56–4.55 (m, 1H, H3); 4.52–4.50 (m, 2H, CH_2_); 4.03 (s, 1H, H2); 3.95–3.67 (m, 4H, H1 + H6); ^13^C NMR (125 MHz, DMSO-*d*6): *δ* (ppm) 167.00 (COOH); 161.55 (C7); 138.02 (Ca); 131.23 (C9); 128.32 (Cb); 127.72 (Cc); 127.61 (C10); 123.12 (Cd); 114.80 (C8); 85.93 (C3); 82.78 (C2); 80.27 (C4); 77.22 (C5); 72.48 (C1); 70.42 (C6); 70.99 (CH_2_); IR: cm^−1^ 2950, 2555, 1674, 1596, 1244, 1170, 1083, 841; *T*_m_ = 148–156 °C.

##### 2.2.6.2. 4-(((3R,3aR,6R,6aR)-6-(benzyloxy)hexahydrofuro[3,2-b]furan-3-yl)oxy)benzoic acid (**2d**)

White powder, 90% yield; ^1^H NMR (500 MHz, DMSO-*d6*): *δ* (ppm) 7.87–7.83 (m, 2H, H9); 7.39–7.27 (m, 5H, Hph); 7.08–7.07 (m, 2H, H8); 4.99–4.90 (m, 2H, H4 + H5); 4.56–4.48 (m, 3H, H3 + CH_2_); 4.05–3.75 (m, 5H, H2 + H1 + H6); ^13^C NMR (125 MHz, DMSO-*d*6): *δ* (ppm) 166.85 (COOH); 161.45 (C7); 137.95 (Ca); 131.10 (C9); 128.19 (Cb); 127.59 (Cc); 127.48 (Cd); 123.10 (C10); 114.71 (C8); 85.88 (C3); 82.76 (C2); 80.17 (C4); 77.17 (C5); 72.42 (C1); 70.29 (C6); 70.21 (CH_2_); IR:cm^−1^ 2950, 2655, 1676, 1596, 1254, 1170, 1082, 850; *T*_m_ = 137–148 °C.

##### 2.2.6.3. 4-(((3R,3aR,6S,6aR)-6-hydroxyhexahydrofuro[3,2-b]furan-3-yl)oxy)benzoic acid (**1g**)

The general debenzylation method was applied to **1d** (1 g, 2.8 mmol) with 20% of Pd/C (0.2 mg) in a mixture of CH_2_Cl_2_/EtOH (15 mL/5 mL) and was reduced under hydrogen pressure (6 bars) at 40 °C.

White powder, 75% yield; ^1^H NMR (500 MHz, DMSO-*d6*): *δ* (ppm 7.82–7.80 (d, 2H, H9, *J* = 8.8 Hz); 7.03–7.02 (d, 2H, H8, *J* = 8.8 Hz); 4.96–4.93 (m, 1H, H4); 4.79–4.77 (t, 1H, H5, *J* = 5 Hz); 4.36–4.34 (t, 1H, H3, *J* = 4.9 Hz); 4.11–4.08 (m, 1H, H2); 4.07–4.04 (dd, 1H, H1 + H6, *J* = 9.1 Hz, *J*’ = 6.3 Hz); 3.80–3.77 (dd, 1H, H1 + H6, *J* = 9 Hz, *J*’ = 6.5 Hz); 3.74–3.71 (t, 1H, H1 + H6, *J* = 7.6 Hz, *J*’ = 7.6 Hz); ^13^C NMR (125 MHz, DMSO-*d6*): *δ* (ppm) 167.39 (COOH); 160.23 (C7); 129.21 (C9); 126.67 (C10); 114.35 (C8); 81.78 (C3); 79.72 (C4); 77.33 (C5); 71.95 (C2); 71.56 (C1); 70.62 (C6); IR :cm^−1^ 3429, 2937, 16,741,598, 1425, 1296, 1244, 1084, 927, 841; *T*_m_ = 152–175 °C.

#### 2.2.7. Synthesis of acid chloride

##### 2.2.7.1. 4-(((3R,3aR,6S,6aR)-6-(benzyloxy)hexahydrofuro[3,2-b]furan-3-yl)oxy)benzoylchloride (**1h**)

**1d** (1.126 mmol, 0.3 g) was refluxed in a mixture of freshly distilled thionyl chloride (1.71 mmol, 0.124 mL) dry chloroform (1 mL), and a few drops of DMF until a clear solution was obtained. The reaction mixture was then concentrated for 1 h under vacuum, the residue was diluted with 5 mL of dry toluene and concentrated again. This procedure was repeated twice to remove residual thionyl chloride. Finally, the product was crystallized from its concentrated toluene solution. Brown solid, 63% yield; ^1^H NMR: (500 MHz, DMSO-*d6*): *δ* (ppm): 7.87–7.71 (m, 2H, H9); 7.34–7.28 (m, 5H, Hph); 7.15–7.05 (m, 2H, H8); 5.00–4.91 (m,1H, H5 + H4); 4.55–4.54 (m, 1H, H3); 4.51–4.50 (m, 2H, CH_2_); 4.03 (s, 1H, H2); 3.95–3.67 (m, 4H, H1 + H6); ^13^C NMR: (125 MHz, DMSO-*d6*): *δ* (ppm): 167.05 (COCl); 161.58 (C7); 138.03 (Ca); 134.07 (C10); 131.27 (C9); 128.34 (Cb); 127.73 (Cc); 127.62 (Cd); 114.81 (C8); 85.98 (C3); 82.84 (C2); 80.32 (C4); 77.26 (C5); 72.52 (C1); 70.46 (C6); 70.31 (CH_2_).

#### 2.2.8. Synthesis of ester

##### 2.2.8.1. Methyl4-(((3R,3aR,6S,6aR)-6-(benzyloxy)hexahydrofuro[3,2-b]furan-3-yl)oxy)benzoate (**1e**)

The same procedure of the acid chloride was applied in the presence of methanol (10 mL). White powder, 77% yield; ^1^H NMR (500 MHz, CDCl_3_): *δ* (ppm) 8.07–7.77 (m, 2H, H9); 7.40–7.28 (m, 5H, Hph); 7.06–6.99 (m, 2H, H8); 4.98–4.95 (m, 1H, H5); 4.87–4.83 (m, 1H, H4); 4.65–4.63 (m, 1H, H3); 4.60–4.58 (m, 2H, CH_2_); 4.20–3.94 (m, 8H, H2 + CH_3+_H1 + H6); ^13^C NMR (125 MHz, CDCl_3_): *δ* (ppm) 171.09 (COOMe); 161.00 (C7); 137.48 (Ca); 132.12 (C10); 129.30 (C9); 128.47 (Cb); 127.87 (Cd); 127.69 (Cc); 115.00 (C8); 86.72 (C3); 83.24 (C2); 80.85 (C4); 77.83 (C5); 73.32 (C1); 71.44 (CH_2_); 70.72 (C6); 60.34 (CH_3_); *T*_m_ = 125–145 °C.

##### 2.2.8.2. Methyl 4-(((3R,3aR,6S,6aR)-6-hydroxyhexahydrofuro[3,2-b]furan-3-yl)oxy)benzoate (**1f**)

The general debenzylation method was applied to **1e** (0.81 mmol, 0.3 g) with 20% of Pd/C (0.6 g) in a mixture of CH_2_Cl_2_/EtOH (5 mL/5 mL), and was reduced under hydrogen pressure (6 bars) at 40 °C.

White powder, 71%yield; ^1^H NMR (500 MHz, DMSO-*d6*): *δ* (ppm) 7.87–7.82 (m, 2H, H9); 7.15–7.03 (m, 2H, H8); 5.19–5.16 (m,1H, H5); 4.97–4.95 (m, 1H, H4); 4.9–4.87 (m, 1H, H3); 4.35 (s, 1H, H2); 4.11–3.64 (m, 7H, CH_3+_H1 + H6); ^13^C NMR: (125 MHz, DMSO-*d6*): *δ* (ppm): 170.00 (COOMe); 160.09 (C7); 131.66 (C10); 129.69 (C9); 114.86 (C8); 88.45 (C3); 79.70 (C4); 79.14 (C5); 78.88 (C2); 78.61 (C1); 75.11 (C6); 69.83 (CH_3_).

### 2.3. Polymer synthesis

The substrate **1g** (1.88 mmol, 0.5 g) was charged into a cylindrical glass reactor and stirred magnetically. The reaction mixture was stirred at 150 °C for 1 h without vacuum and a homogeneous medie was obtained. The temperature was then increased gradually to 200 °C, and the polymerization was kept undergo for 2 h under vaccuum. After cooling to room temperature, the crude was dissolved in DMF (3 mL) and precipitated into cold MeOH (10 mL) giving rise to a brown solid. ^1^H NMR: (500 MHz, DMSO-*d6*): *δ* (ppm): 7.92–7.82 (m, 4H, H9,); 7.21–7.07 (m, 4H, H8); 5.31 (s, 1H, H3); 5.22 (s, 1H, H2’); 5.08 (m, 2H, H2 + H5); 5.08–4.94 (m, H4 ‘ + H5’); 4.91–4.89 (m, H3’’); 4.65 (s, 1H, H4); 4.35–4.34 (m, H2”); 4.12–3.95 (m, H1 + H6); 3.91–3.63 (m, H1’ + H6’ + H1’’ + H6’’); ^13^C NMR: (125 MHz, DMSO-*d6*): *δ* (ppm): 167.44 (C11’); 166.95 (C11’’); 164.64 (C11); 162.17 (C7); 162.05 (C7’’); 161.45 (C7’); 131.31 (C10); 131.18 (C9); 129.24 (C9’); 121.62 (C10’’); 121.45 (C10’); 115.00 (C8); 114.77 (C8’’); 114.37 (C8’); 88.61 (C3); 85.96 (C4); 80.53 (C3’’); 79.98 (C5); 78.05 (C4’); 77.40 (C5’’); 77.15 (C5’); 75.24 (C2); 74.94 (C2’); 72.63 (C1); 70.72 (C6’); 70.11 (C6); IR :cm^−1^ 3404, 2875, 1710, 1599, 1505, 1244, 1186, 767; *T*_m_ = 83–113 °C.

## 3. Results and discussion

The synthesis of the dinitrile monomers was optimized compared to the anterior works.[25] The previous method reported for the dinitrile compound synthesis, described the use of microwave irradiation at 170 °C during 30 min. In our context, the first step was carried out under solvent-free conditions and adapted to the classical heating method at 170 °C which renders it more accessible. The dinitrile was obtained by a S_*N*_Ar reaction between the DAH and *p*-fluorobenzonitrile in basic medium. This method was applied in the absence of any organic solvent, using KOH as the base and 18-crown-6 as the phase transfer agent. After 4 h, the dinitrile compound was recovered by precipitation or by flash chromatography on silica gel. This method was extended to the two other dianhydrohexitols namely Isomannide **2** and Isoidide **3** (Scheme 1). After exact mass confirmation using HRMS analysis, NMR studies were carried out to confirm the structures and highlight the characteristic signal of each dianhydrohexitol type (Figures S2 and S3 in the Supporting Information).

**Scheme 2.**
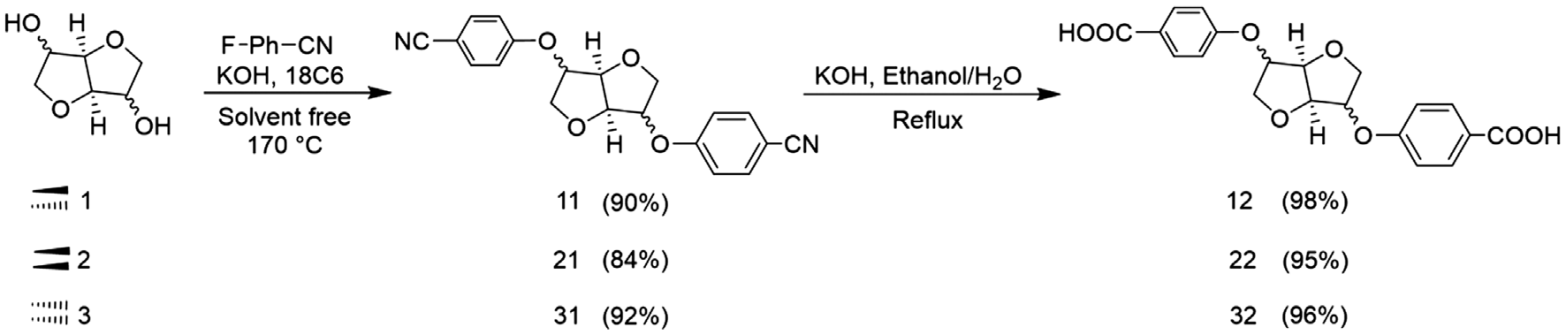
Synthesis of biosourced monomers AA derived from dianhydrohexitols.

We first focused our efforts on the hydrolysis of the dinitrile functions to the corresponding diacids (Scheme 2). Various aqueous nitrile hydrolysis procedures (both acidic and basic) gave unsatisfactory results, since none of the wide range of investigated conditions gave a full conversion of the nitrile groups. Due to the high polarity of mono nitrile-acid intermediate and the targeted diacid, effective separation of the latter from the crude reaction mixture turned out to be challenging. We then investigated the basic hydrolysis in the presence of KOH by using a mixture of EtOH/H_2_O. The diacid was then obtained by precipitation without any further purification and recovered in high purity after several water washings.

**Figure 1.**
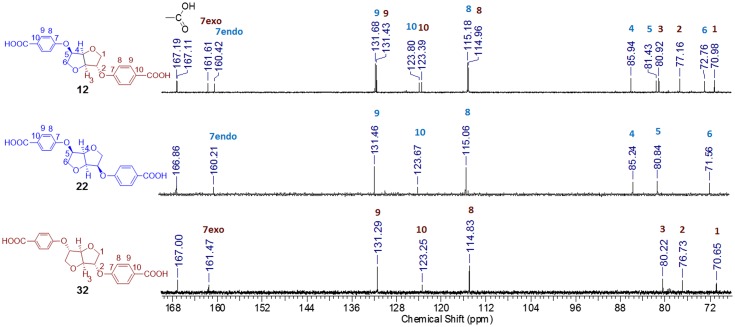
^13^C spectra of diacids **12**, **22** and **32** in DMSO-d6.

Based on our latest results we aimed to demonstrate the effect of the starting DAH stereochemistry on the products properties. The comparison of the DAH core signals (Figure 1) clearly showed that no epimerization occurred during all the process. Indeed, symmetrical Isomannide and Isoidide exhibited three types of signals, whereas Isosorbide patterns showed six. In the same trend, under the influence of the *endo*/*exo* alternation of the signals for the latter, the carbons directly attached to the hydroxyl functions split into two signals (C7: 160.4 and 161.6 ppm). This overlaid allowed us as well to attribute without any doubt.

**Figure 2.**
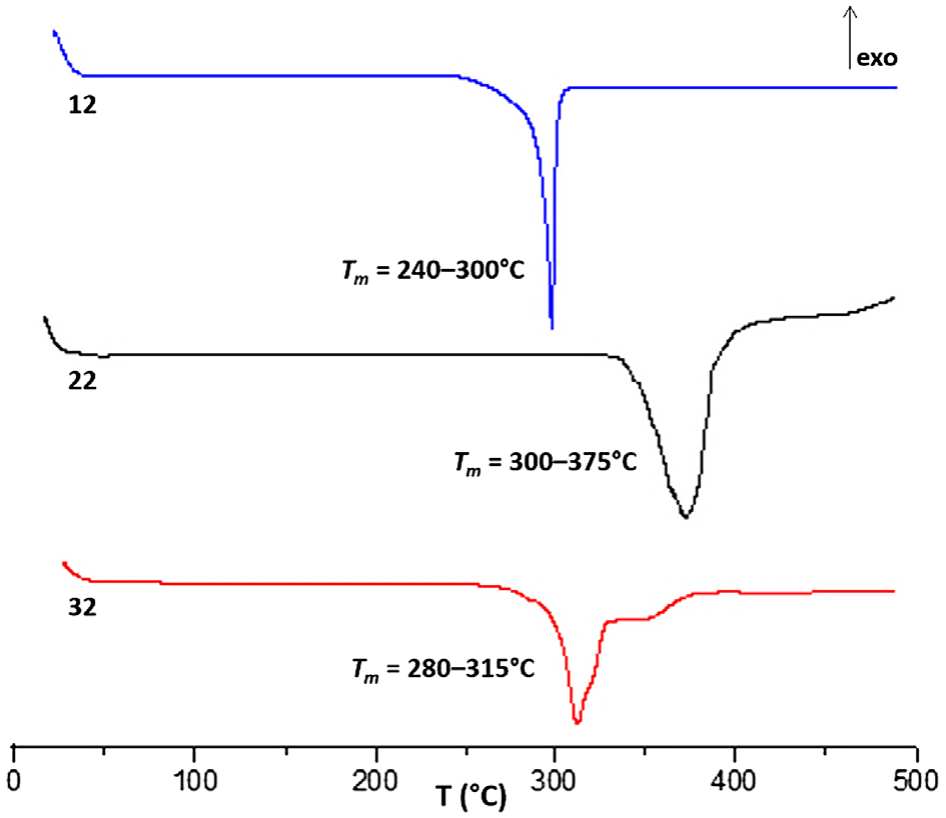
DSC curves of **12**, **22** and **32** measured between 17 and 500 °C at 10 °C/min heating.

Thermal analysis of these new diacids (Figure 2) showed interestingly high melting ranges (240–375 °C) which make them being potential candidates for polymers with high thermal performance. As a trend, it seemed that stereochemistry of the starting DAH influences the melting point, the Isomannide homologue **22** presenting the highest *T*_m_ range. This could probably be explained by an additional intramolecular association favored by the *endo* orientation of both benzoic acid moieties .

**Figure 3.**
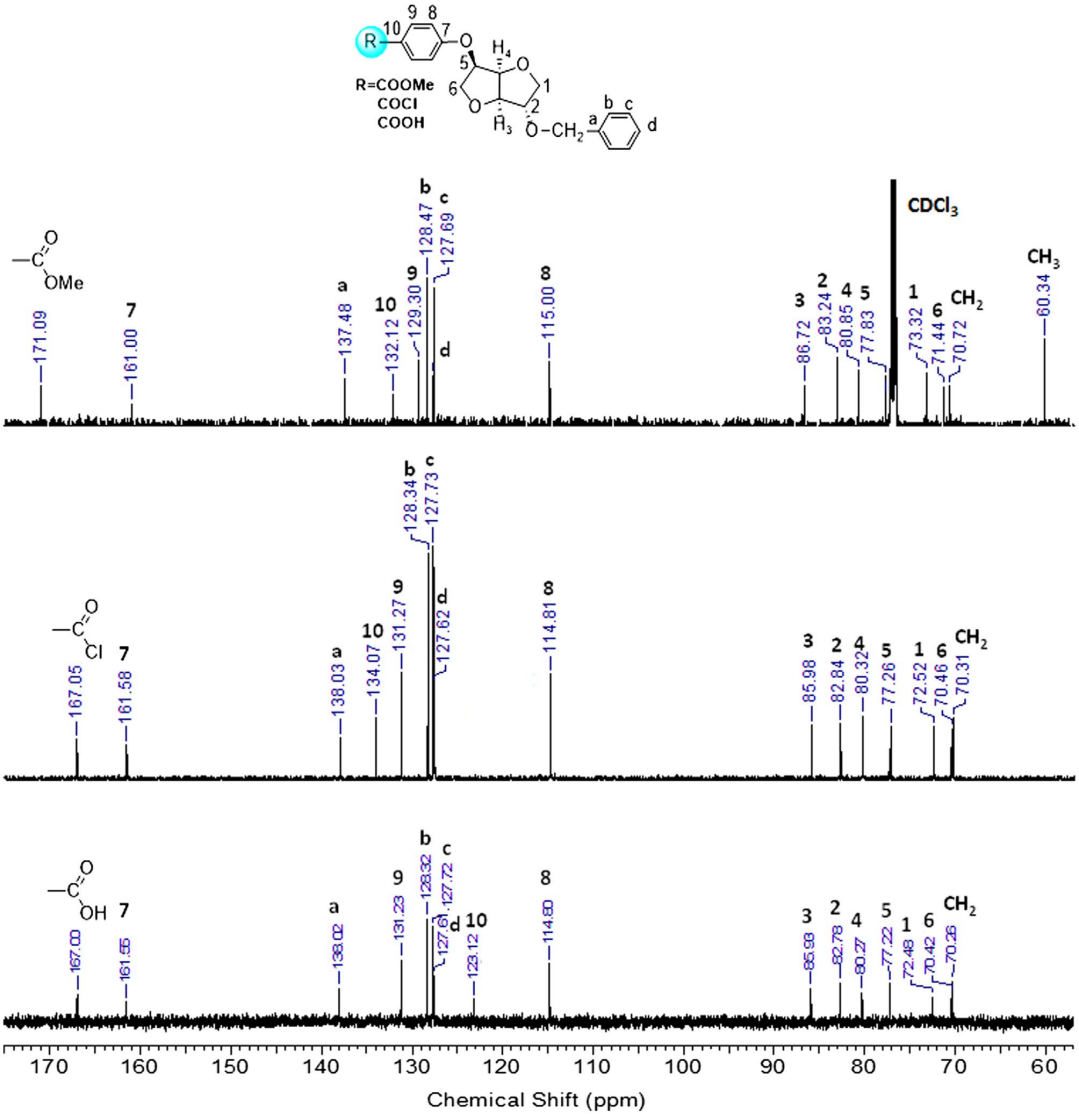
^13^C spectra of **1g** (A) (DMSO-d6), **1h** (B) (DMSO-d6) and **1e** (C) (CDCl3).

**Figure 4.**
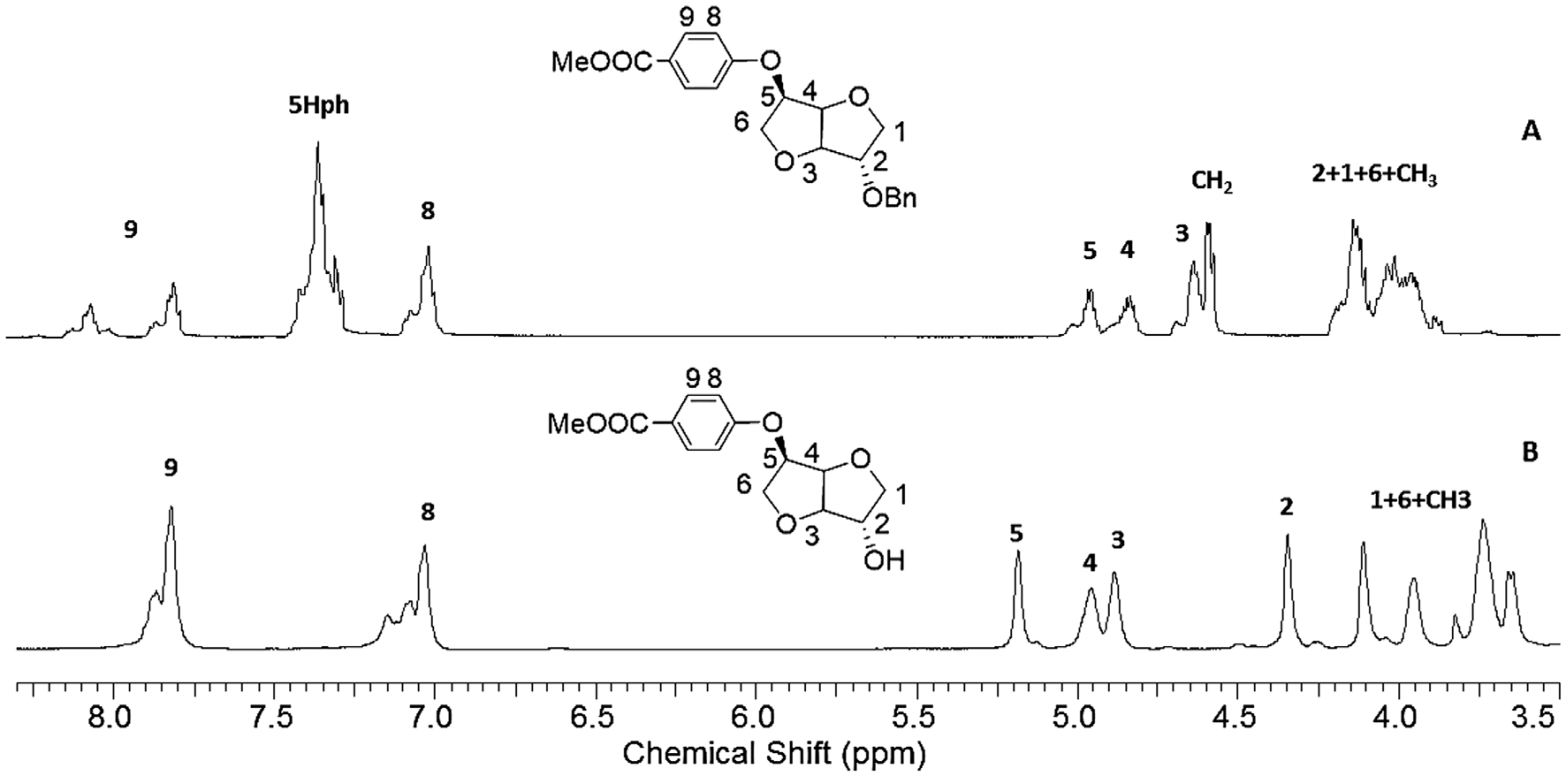
^1^H spectra of **1e** (A) in DMSO-d6and **1f** (B) in CDCl3.

**Figure 5.**
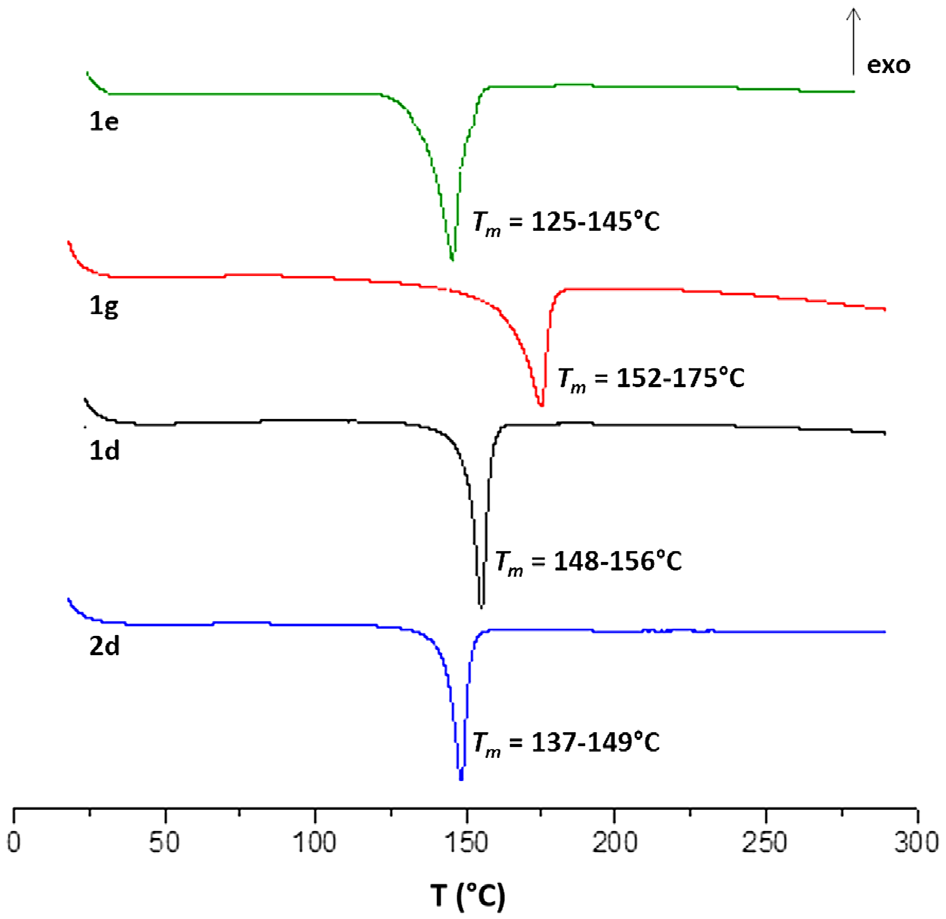
DSC curves of **2d**, **1d**, **1g** and **1e** measured between 18 and 300 °C at 10 °C/min heating.

**Figure 6.**
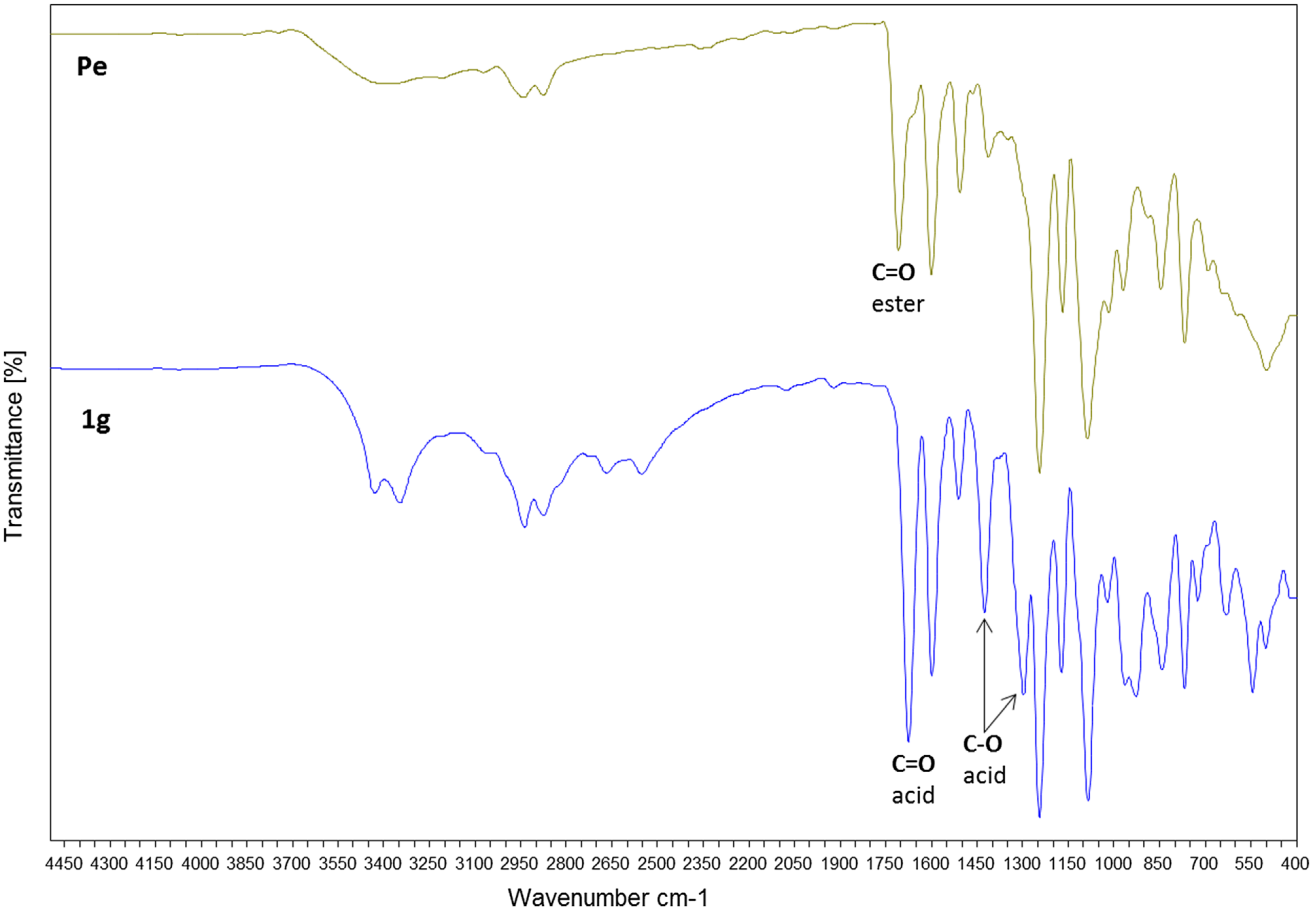
FT-IR spectra of **1g** and **Pe** recorded at room temperature.

**Figure 7.**
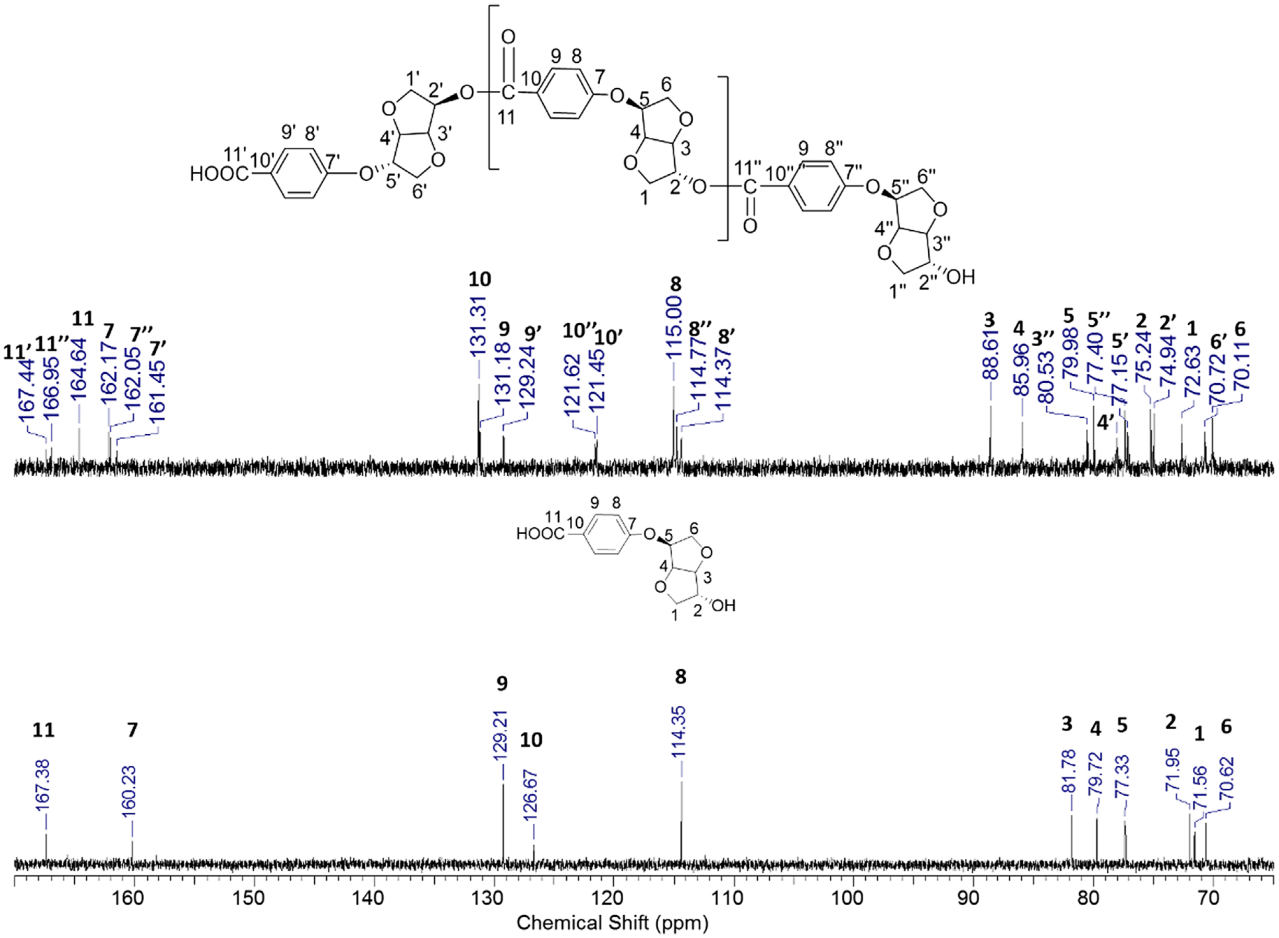
^13^C spectra of the monomer **1g** and the polymer **Pe** in DMSO-d6.

**Figure 8.**
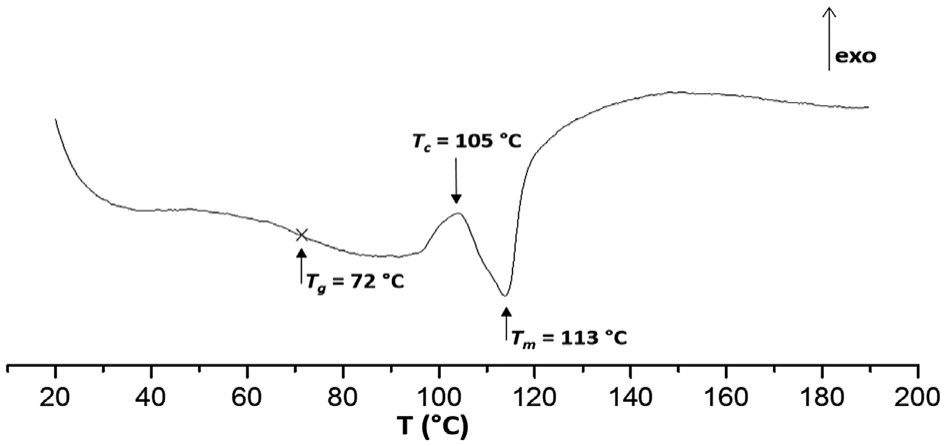
DSC curves of **Pe** measured between 18 and 250 °C at 10 °C/min heating.

**Scheme 3.**
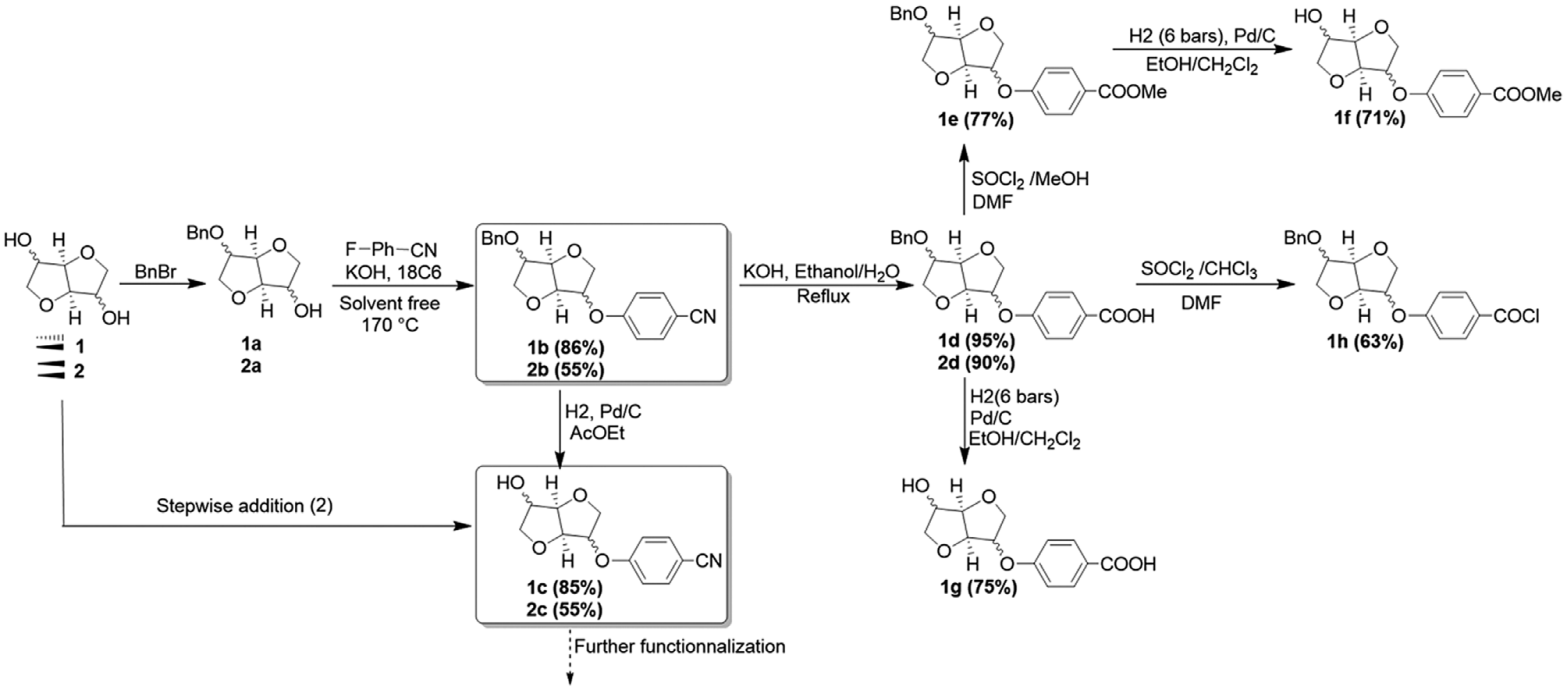
Synthesis of biosourced monomers AB derived from dianhydrohexitols.

In order to extend the viability and flexibility of this approach, we decided to develop a set of self polymerizable AB type monomers. It is well established that such monomers present the benefit of avoiding side reaction as well as an accurate control of the stoichiometry. The monoarylation on Isosorbide requires the preliminary protection of one hydroxyl function due to the difference of reactivity of both OH functions. Due to the stereochemistry and configurational restrictions, the two hydroxyl functions of Isosorbide react differently.[26] The strategy was then based on a prior protection of the *exo* hydroxyl function with monobenzylation,[27] followed by a nucleophilic substitution on the free left hydroxyl group. The obtained monophenylnitrile intermediates **1b/2b** represents a new chemical platform (Scheme 3). In fact, several pathways could be imagined starting from this intermediate. After debenzylation, it could afford the deprotected alcohol **1c** or **2c**. The latter would allow selective modification on the alcohol side considering the nitrile function as an acid precursor. Based on our recent works regarding selective mono arylation on symmetrical Isomannide **2**,[22] a similar strategy was considered. Lowering the temperature to 150 °C and using 0.5 eq of arylating agent afforded the desired compound **2c** in only one step with 84% yield (Table 1, entry 12).

**Table 1.** Optimisation of reaction parameters and yields of products.

Entry	Reagent	Quantity of reagent (g)	*T* (°C)	Addition mode	Reaction time (h)	Results^a^
1		0.1	100	Simultaneous	24	44
2		0.1	150	Simultaneous	18	55
3		0.5	150	Simultaneous	24	36
4		1	170	Simultaneous	24	40
5	1a	1	170	Simultaneous	4	86
6		3	170	Simultaneous	4	84
7		5	170	Simultaneous	4	81
8		10	170	Simultaneous	4	82
9		0.1	170	Simultaneous	4	57
10	2a	1	170	Simultaneous	4	55
11		6	170	Simultaneous	4	52
12	2	0.1	150	Fractionwise	5	84%mono/7%di

^a^ Yields in isolated products.

Since the protected nitrile compound **1b**/**2b** is the key intermediate in our strategy we explored several ways to optimize the isolated yield (Scheme 3). To this end, reaction parameters such as temperature, time, and reactant ratios were examined. Monobenzylated Isosorbide and *p*-fluorobenzonitrile were reacted, in solvent-free conditions, in presence of KOH as a base and 18-Crown-6 as a phase-transfer agent. After precipitation in cold methanol or chromatography purification, the protected nitrile compound **1b**/**2b** was isolated as a yellow crystalline powder with high yield (84%). A scale up to 10 g could be achieved conveniently without yield loss (Table 1, entry 8).

Further steps to the desired AB monomer deserved some interesting comments. Indeed, we figured out that a preliminary deprotection of the alcohol prevented the hydrolysis of the nitrile function to take place. In order to overcome this problem, we focused our efforts on working with the protected monomer **1b** or **2b**. The hydrolysis step was then carried out at the first place to afford **1d** or **2d** with high yield (90–95%). In order to render these AB monomers the most versatile possible, we explored several ways to achieve their synthesis. Starting from the protected acid reactant **1d** (Figure 3(A)), a debenzylation was conducted in a mixture of EtOH and CH_2_Cl_2_ to afford the product **1g** with 75% yield. On the other hand, the ester analogue was synthesized starting from **1d** in one pot via joint addition of SOCl_2_/MeOH leading to **1e** with 77% yield (Figure 3(C)). The obtained protected ester revealed more soluble. The debenzylation led to **1f** with 71% yield. The disappearance of signals due to the benzyl group clearly indicated the occurrence of the reaction (Figure 4). Working in a mixture of SOCl_2_/CHCl_3_ gave rise to the protected acyl chloride **1 h** with 63% (Figure 3(B)).

Worth noting, as mentioned above, the stereochemistry influences drastically the thermal behavior of the DAH monomers (Table 2). First, comparing Isosorbide based protected acid **1d** with his homologue isoidide **2d** showed a slightly higher *T*_m_. When **1d** was deprotected to **1g** the *T*_m_ increased probably due to the additional OH bonding of the free hydroxyl (Figure 5). Second, Jaffe and al. reported a melting point for Isoidide based phenyl ester analogue of 117 °C,[24] whilst the corresponding protected Isosorbide analogue **1e** melts in the range of 125–145 °C.

Having all these appealing monomers in hand, we aimed to show a proof of concept starting from the Isosorbide based acid analogue **1g** considering it as the most promising monomer due to its commercial availability and straight forward synthesis. We then investigated the potential use of these monomers as building blocks in polymer materials synthesis. In addition, to the utility of AB monomers type in terms of accurate stoichiometry in our case, we expect them to give rise to a polymer with stereoregular sequences which would induces some crystallinity ordering. We carried out the polymerization of **1g** in bulk. The reaction media was heated at 150 °C to ensure the melting of **1g** and then increased to 200 °C to carry on the polycondensation. A precipitation of the crude material that is previously dissolved in DMF was conducted in cold MeOH leading to a solid material. FTIR and NMR analysis were conducted in order to follow the polymerization.

FTIR analysis (Figure 6) evidenced the successful polymerization reaction. In fact, bands at 1676, 1425 and 1296 cm^−1^ related to the C–O–C stretching vibration of the acid function disappeared as well as the large band at 3400 cm^−1^.[28,29] A new band corresponding to the neoformed ester bond was observed at 1710 cm^−1^.

^13^C NMR spectra of polyester **Pe** overlaid with starting monomer **1g** are shown in Figure 7. We observed the presence of signals with high intensity and others with low ones corresponding to the carbons from the main chain of the polymer and the end chain moieties, respectively. Looking carefully to the ^13^C NMR spectra superposition of monomer **1g** and polymer **Pe,** we noticed the appearance of newly formed carbonyl signal relative to ester group C11 of the polymer backbone, besides signals C11’ and C11” corresponding to the acid and to the ester functions in the end chain respectively. Moreover, the spectrum showed novel signals owing to the main chain polymer. Several signals were greatly shifted due to the change in the chemical neighborhood. This shift was different from one carbon to another, depending on its location along the polymer chain. Especially, signals corresponding to C3 and C4 in the Isosorbide backbone were detected respectively at 88.6 and 85.7 ppm. This remarkable shift was observed in previously reported Isosorbide based polyester terephthalates.[8] C1 appeared at 72.6 ppm. C2, which is directly linked to the hydroxyl function, shifted from 71.9 to 75.2 ppm. We observed also new signals corresponding to aromatic C9, C8, and C10. The latter remarkably shifted from 126 ppm in the monomer to 131 ppm due to the neoformed ester bond.

Thermal properties of the polymer **Pe** were next investigated by DSC analysis (Figure 8). While **1g** had a melting point *T*_m_ = 152–175 °C (Figure 5), we observed for **Pe** a melting point range of *T*_m_ = 83–113 °C. As expected, this strategy gratefully afforded semicrystalline polymer starting from Isosorbide ether benzoic acid, which have never been reported. Anterior works reporting the direct use of Isosorbide with terephthaloyl chloride gave rise to random *exo*/*endo* sequences alternation rendering the obtained polymer amorphous.[30,31]

## 4. Conclusion

New biosourced monomers bearing nitrile, acid, ester, and acid chloride functionalities were synthesized based on Isosorbide and its isomers Isomannide and Isoidide. The aim of this work was to set up a wide range of appealing and promising precursors starting from a renewable platform for biobased AA and AB chemicals derived. The process involved a first solvent-free S_*N*_Ar step on *p*-fluorobenzonitrile followed by an optimized quantitative hydrolysis of the nitrile function. The stereochemistry proved to be of great impact on the thermal properties of these monomers which melted in a range from 240 to 375 °C for AA type diacids and from 80 to 175 °C for AB type monomers. For the latter, a comprehensive synthesis strategy was undertaken in order to forecast the scope of obtaining a set of versatile AB type precursors bearing different functionalities. For the first time, polymer based on the novel Isosorbide ether benzoic acid has been successfully synthesized via melt polymerization. The obtained polymer revealed semicrystalline thanks to the stereoregular arrangement provided by the AB pattern .

**Table 2.** Thermal properties of monomers AA and AB measured by DSC.

DAH	Product	Yield (%)	*T*_m_ (°C)
**1**	11	90	130–137
	12	98	240–300
	1b	86	84–86
	1c	85	79–81
	1d	95	148–156
	1e	77	125–145
	1f	71	–
	1g	75	152–175
	1h	63	–
**2**	21	84	178–184
	22	95	280–315
	2b	55	84–85
	2c	84	119–125
	2d	90	137–149
**3**	31	92	160–165
	32	96	300–375

Nevertheless, challenges remain to be overcome regarding the processing of such polymers and the improvement of their aspects. These new monomers showed high thermal stability which can contributes to limit sublimation and reduce the color production in melt polymerizations. Moreover, the isosorbide derived AB monomers, thanks to their controlled stoichiometry and stereochemistry, could give rise to polymers with high molecular weights and *T*_g_ thus increasing thermal stability of polycondensates which is important for polyesters processing such as bottles, films, sheets, and fibers. AA type monomers which showed high thermal stability, could be successfully incorporated into commercial available polymers like PET to afford copolymers with enhanced thermal properties and an inherent biodegradability to help overcoming current environmental problems.

## Disclosure statement

No potential conflict of interest was reported by the authors.

## Funding

This work was supported by the Ministry of Higher Education, Scientific Research, Tunisia, together with EGIDE in the framework of the CMCU project [Project PHC Utique 13G/1211].

## Supplemental data

Supplemental data for this article can be accessed here http://dx.doi.org/10.1080/15685551.2016.1239175

## Supplementary Material

TDMP_1239175_Supplementary_Materials.pdf
