# Supplementary material for: New biosourced AA and AB monomers from 1,4:3,6-dianhydrohexitols, Isosorbide, Isomannide, and Isoidide
Source: Des Monomers Polym. 2016 Oct 23;20(1):221–33. doi: 10.1080/15685551.2016.1239175 (PMC5812125; doi:10.1080/15685551.2016.1239175)
Supplement: TDMP_1239175_Supplementary_Materials.pdf [file TDMP_A_1239175_SM4339.pdf]

Supporting Information

New biosourced AA and AB monomers from 1,4:3,6-dianhydrohexitols, Isosorbide, Isomannide and Isoidide

Asma Saadaoui[a,c,d], Raouf Medimagh[a*], Sylvain Marque[b], Damien Prim[b] , Saber Chatti[c], Herve Casabianca[c]&Mongia Said Zina[d]

[a]Institut National de Recherche et d'Analyse Physico-chimique (INRAP), Laboratoire des Substances Naturelles (LR10INRAP02), Sidi ThabetBiotechpole, Ariana 2020, (Tunisia).

[b]Université de Versailles-St-Quentin (UVSQ), Institut Lavoisier de Versailles (ILV) UMR CNRS 8180, 45 Avenue des Etats-Unis, 78035 Versailles Cedex, France

[c]Université de Lyon 1, Institut des Sciences Analytiques, UMR5280, CNRS, ENS-Lyon, 5 rue de la Doua, F-69100 Villeurbanne (France).

[d]Faculté des Sciences de Tunis, Université de Tunis El Manar 2092 Tunis (Tunisia).

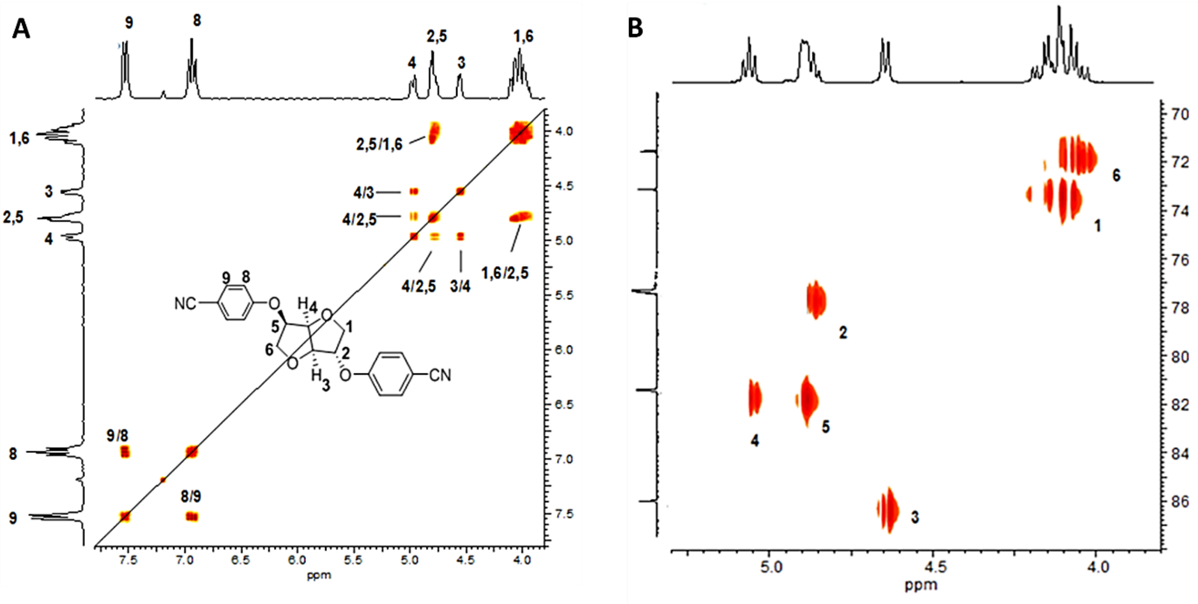

Figure S1.300 MHz 2D correlation spectra (A) COSY and (B) HSQC of **11**

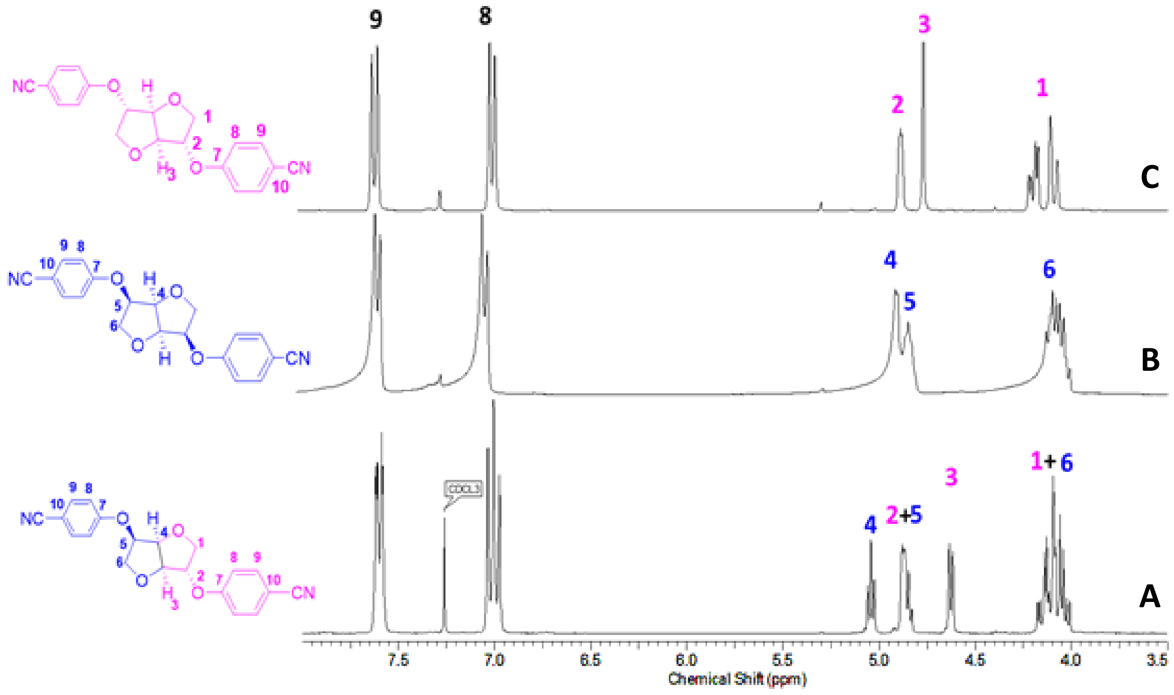

Figure S2.^{1}H spectra of 11 (A), 12 (B) and 13 (C).

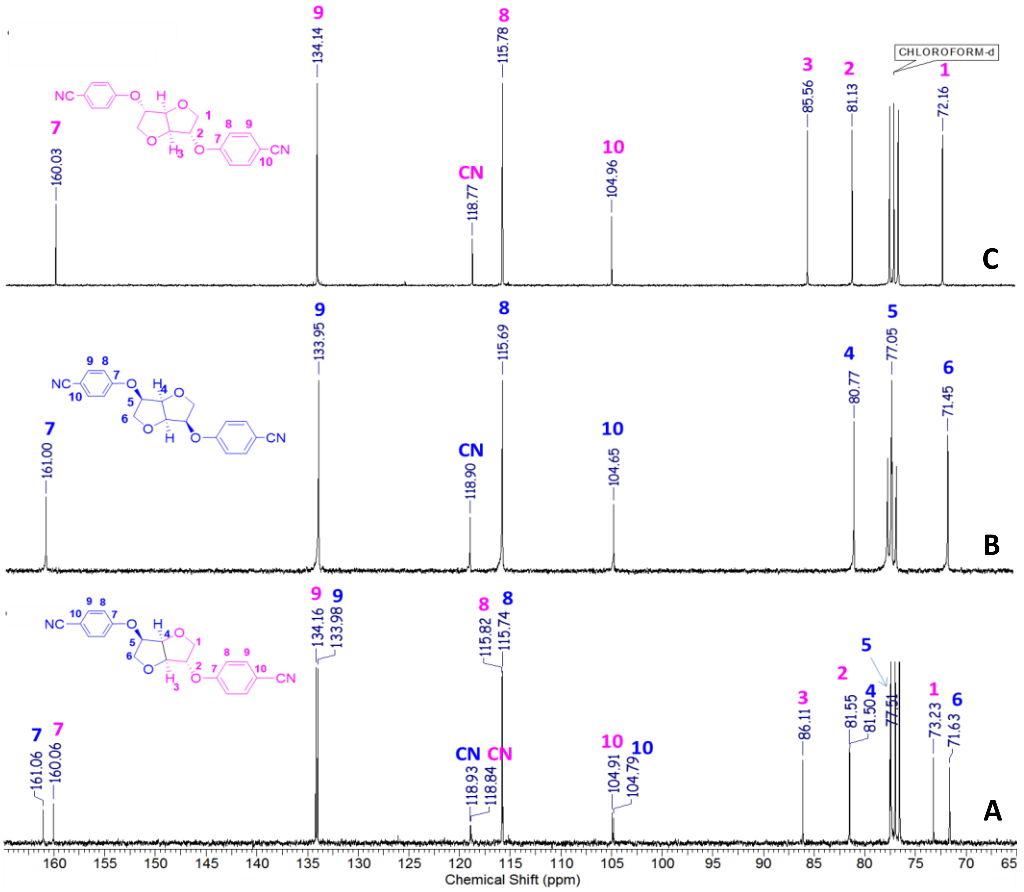

Figure S3.^{13}C spectra of 11 (A), 12 (B) and 13 (C)

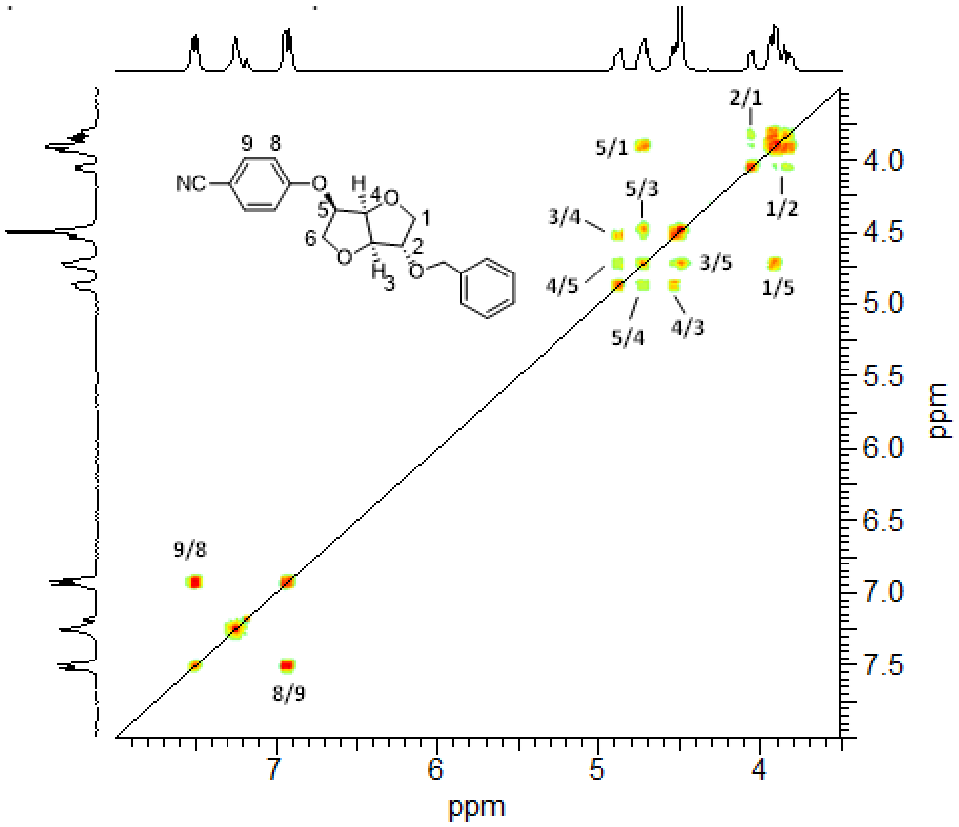

Figure S4. 300 MHz correlation spectrum (COSY) of **1b.**

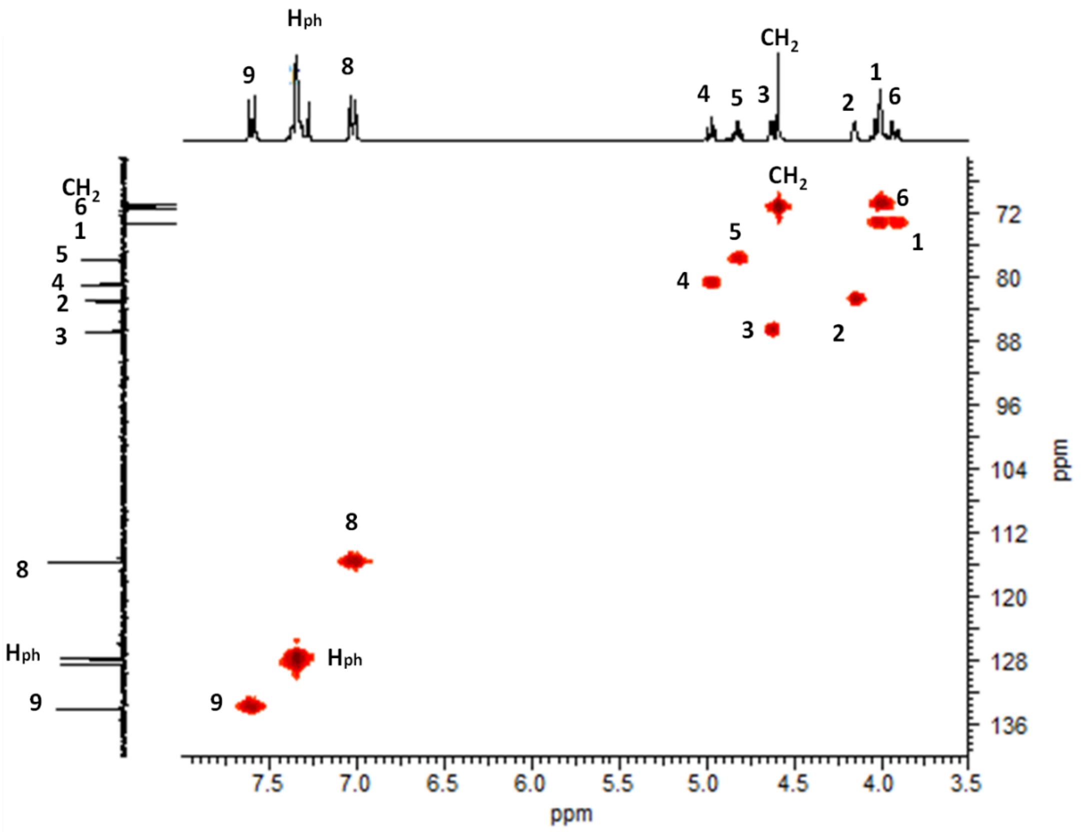

Figure S5. 300 MHz correlation spectrum (HSQC) of **1b**

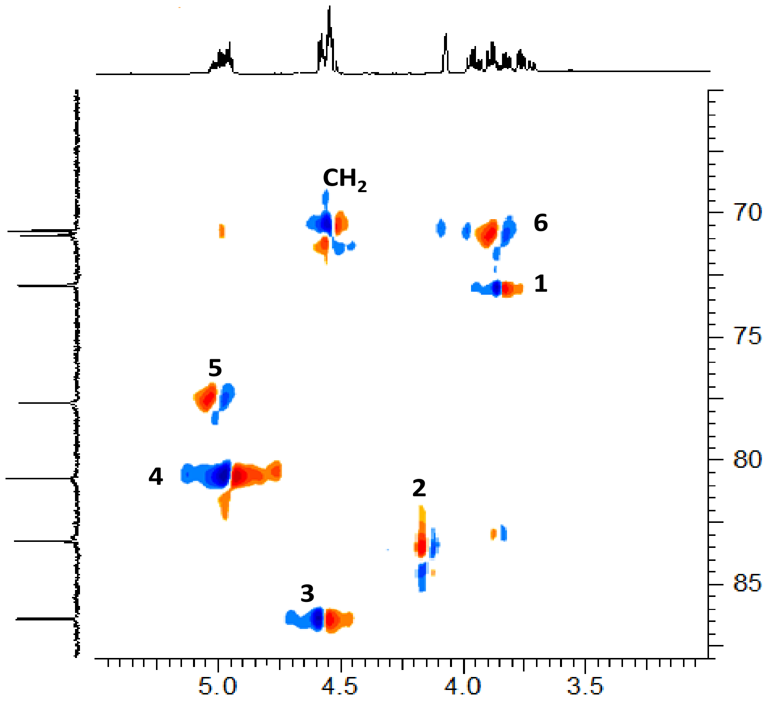

Figure S6. Zoom of 500 MHz correlation spectrum (HSQC) of **1h**

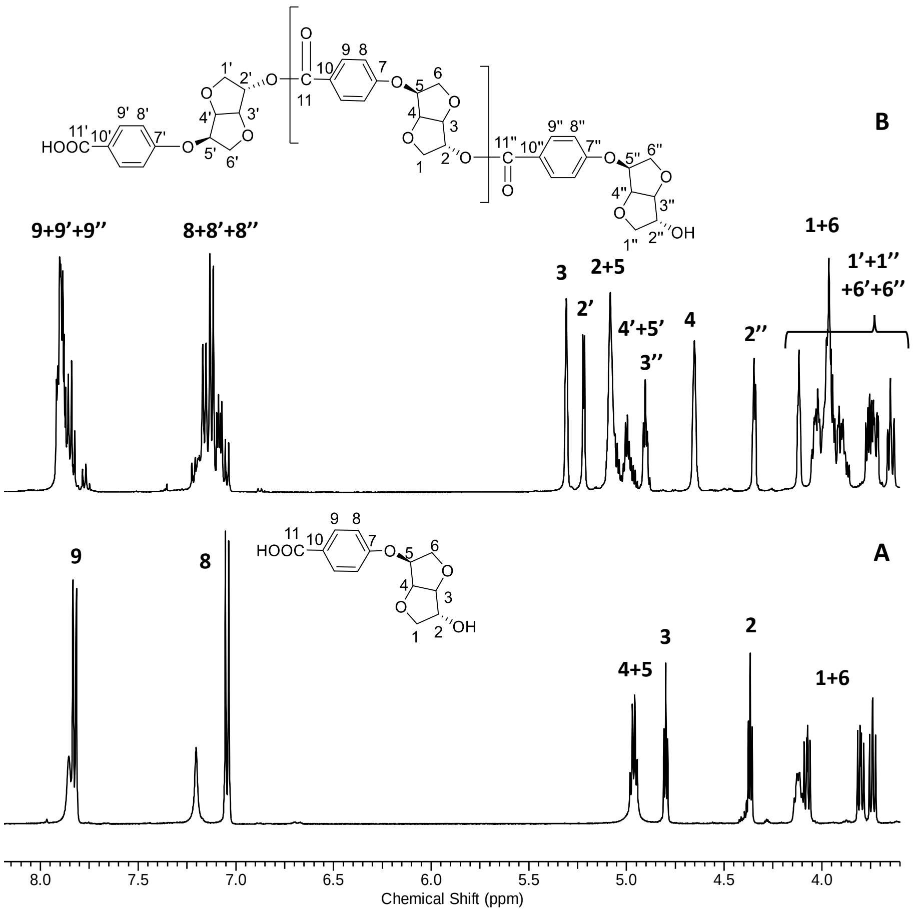

Figure S7.^{1}H spectra of **1g** (A) and **Pe**(B) [DMSO-*d6*, 500 MHz]
